# The impact of antigenic molecular mimicry on anti-cancer T-cell immune response

**DOI:** 10.3389/fonc.2022.1009247

**Published:** 2022-10-18

**Authors:** Maria Tagliamonte, Luigi Buonaguro

**Affiliations:** Innovative Immunological Models Unit, Istituto Nazionale Tumori - IRCCS - “Fond G. Pascale”, Naples, Italy

**Keywords:** molecular mimicry, cross-reacting T cells, anti-cancer immune response, cancer vaccine, microbiota, virus, tumor antigen

## Abstract

Individuals are exposed to intracellular pathogens (i.e. viruses and intracellular bacteria) and intestinal microbiota, collectively microorganisms (MOs), which enter the body during the host’s lifetime. Altogether, MOs are a natural source of non-self antigens (MoAs) expressed by host’s cells in the context of the HLA class I molecules, inducing a wide pool of specific memory CD8^+^ T cell clones. Such MoAs have been shown in selected cases to share sequence and structural homology with cellular self-antigens (molecular mimicry), possibly inducing autoimmune reactions leading to autoimmune diseases (ADs). We have recently shown that a molecular mimicry may be found also to self-antigens presented by cancer cells (i.e. tumor-associated antigens, TAAs). Consequently, memory CD8^+^ T cell clones specific for the MoAs may turn out to be a natural “anti-cancer vaccination” if a nascent tumor lesion should express TAAs similar or identical to MoAs. We postulate that selecting MoAs with high homology to TAAs would greatly improve the efficacy of cancer vaccines in both preventive and therapeutic settings. Indeed, non-self MoAs are potently immunogenic because not affected by central immune tolerance. Unravelling the impact of the antigenic molecular mimicry between MoAs and TAAs might pave the way for novel anti-cancer immunotherapies with unprecedented efficacy.

## Introduction

Anti-cancer immune response mostly fails to control tumor growth for the immunosuppressive factors infiltrating the tumor microenvironment (TME) (1). In addition, the target antigens presented by cancer cells are either poorly immunogenic (tumor-associated antigens, TAAs) or passenger mutations lost during tumor evolution (tumor-specific antigens, TSAs) (2, 3).

In particular, TAAs are universal antigens shared by all patients diagnosed with the same malignancy and can be used to develop off-the-shelf cancer vaccines. However, they are wild-type self-antigens overexpressed in tumor cells with variable expression levels in normal cells. Therefore, TAAs are generally characterized by low immunogenicity and specific T cells may have low affinity T cell receptors (TCR) with poor anti-tumor effect (4). Moreover, such T cells might have been removed from the immune repertoire by central and peripheral tolerance to prevent auto-immune reactivity to self-antigens (5).

Consequently, TAA-based clinical trials have shown extremely variable induction of a CTL response with a limited efficacy on clinical outcome (6, 7).

The identification of novel universal shared antigens for development of off-the-shelf cancer vaccines with superior immunogenicity is a primary goal in cancer immunotherapy field.

## Molecular mimicry

The molecular mimicry may provide an important help in such a quest. The homology between microbial and host cell antigens has been shown in several cases. Most importantly, immune tolerance may be broken and antibodies or T cells elicited by a non-self-viral or bacterial antigen can cross-react with the homologous self-host antigen. This would ultimately initiate an autoimmune reactivity which may eventually progress to a disease (8). Indeed, different epitopes binding to the same HLA allele and exposing similar residues or structures can be targeted by the same CD8^+^ T cell receptor (TCR) (9, 10).

Similarly, the homology between microbial and self-host cell antigens overexpressed in tumor cells may provide a rationale for selecting a pool of highly immunogenic non-self antigens able to elicit cross-reacting anti-tumor T cells.

Evidences for molecular mimicry performed by T cells elicited by selected microbial antigens able to cross-react with specific homologous tumor-associated antigens have been reported. In particular, homology between MAGE-A6 and Mycoplasma penetrans HF-2 epitopes (11), Melanoma/Melanocyte-derived Peptide MART-1_(27-35)_ and Herpes simplex virus (HSV) epitopes (12), MAGE-A10 and Cytomegalovirus (CMV) (13). Moreover, tumor-infiltrating lymphocytes exhibit cross-reactivity against non-mutated TMEM161A tumor antigens and Epstein Barr virus (EBV) antigens (14). A recent report showed the T cell cross-reactivity between homologous epitopes contained in the PSMB4 protein and the Siphoviridae phage infecting the enterococcus E. hirae strain (15). Likewise, mutated tumor-specific neoantigens in melanoma have been found sharing homology with bacterial antigens (16).

In this framework of experimental proof on selected tumor and microbial antigens, we have recently searched for a more general evidence for molecular mimicry between TAAs and antigens derived from microorganisms.

## Homology between TAAs and viral-derived antigens

**Figure 1:**
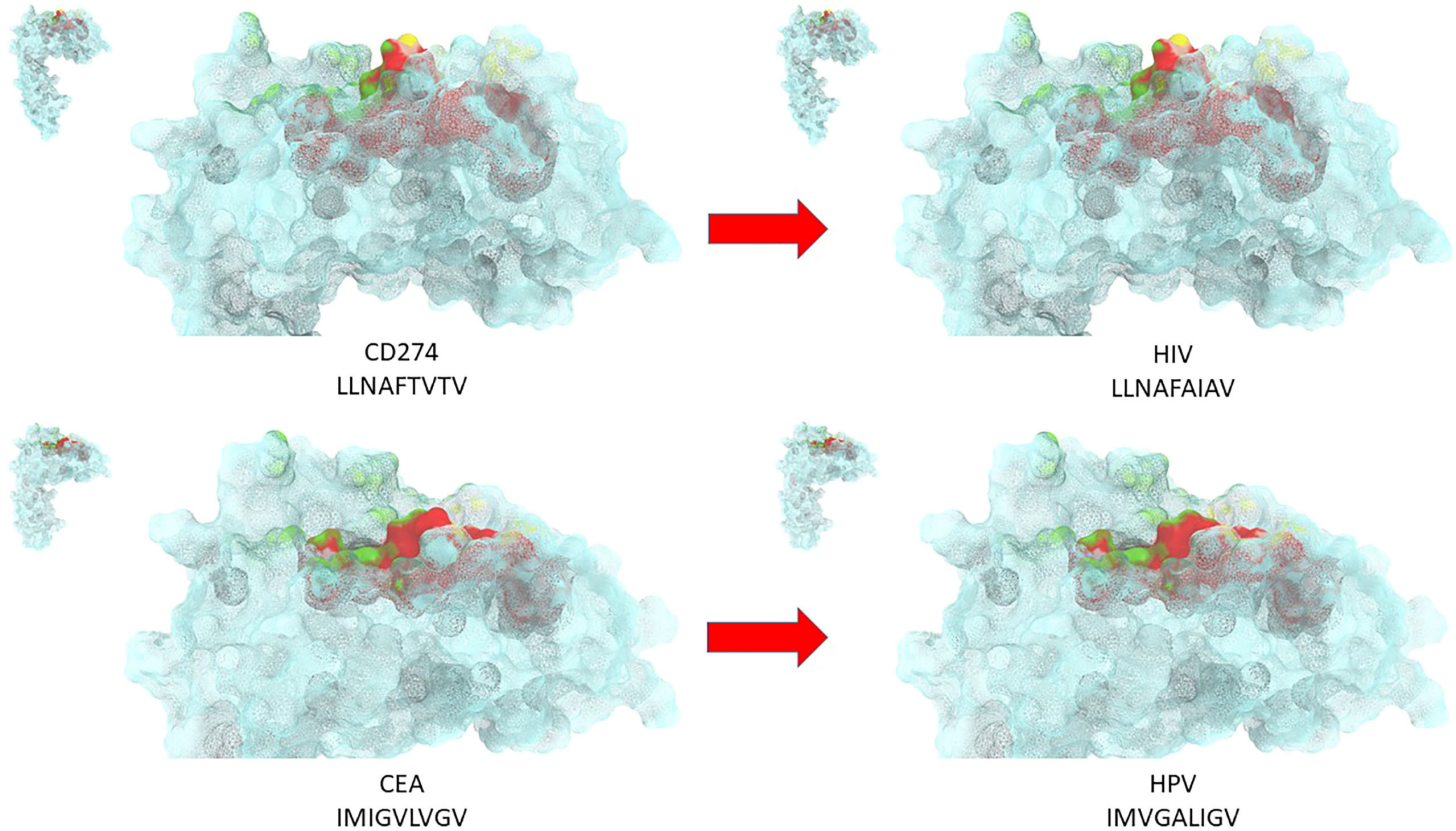
Structural predicted conformation of paired TAA and viral peptides. The conformation of the paired viral and TAA peptides bound to the HLA-A*02:01 molecule is shown. The prediction was performed using as template structure the HTLV-I LLFGYPVYV peptide crystallized with the HLA-A*0201 molecule, the β2 microglobulin, the α and β chains of the TCR (PDB https://www.rcsb.org/structure/1AO7). Green areas = contact points with the TCR α chain; Yellow areas = contact points with the TCR β chain.

All the TAAs from the cancer peptide database (https://caped.icp.ucl.ac.be/Peptide/list) were submitted to BLAST for a protein homology search against viral sequences (Viruses - taxid:10239). In particular, TAAs were selected among those binding to the MHC class-I HLA-A*0101, 0201, 0301 and 2402 alleles, which altogether cover about 50% of the world population (17). 82 viral sequences sharing homology with the TAAs were identified. Surprisingly, the Human Immunodeficiency Virus type 1 (HIV-1) contributed by far with the largest number of viral sequences (36/82). The paired TAA and viral epitopes not only share the same conformation but also similar, if not the same, contact patterns with the HLA-A molecules, as well as the TCR α and β chains. Structural preservation between paired peptides is dependent on conservative amino acid changes at specific positions. This would strongly suggest that, for each pair, the same CD8+ T cell clone may be able to cross-react with both peptides when presented in the context of the HLA-A molecule. Ex vivo immunization experiments confirmed the T cells cross-reactivity with the paired TAA and viral epitopes (17). In addition, we have identified potential novel epitopes binding to the MHC Class-I HLA-A*02:01 and 24:02 alleles as target for innovative HCC-specific immunotherapy strategies (18). Homology with viral epitopes was found, including influenza virus, hepatitis C virus (HCV), hepatitis B virus (HBV), adenovirus, human cytomegalovirus (HCMV) and human calicivirus. Also in this case, the epitope modeling confirmed that the paired TAA and viral epitopes share the same contact patterns with the HLA-A molecules, as well as the TCR α and β chains when conservative changes occur at specific positions of the paired epitopes (**Figure 1**).

Ex vivo binding assays in TAP-deficient T2 cells confirmed the predicted affinity of paired epitopes to the HLA-A*02:01 allele. Moreover, cross-reactive T cell responses against the paired peptides was observed by tetramer staining assay (18).

## Homology between TAAs and microbiota-derived antigens

**Figure 2:**
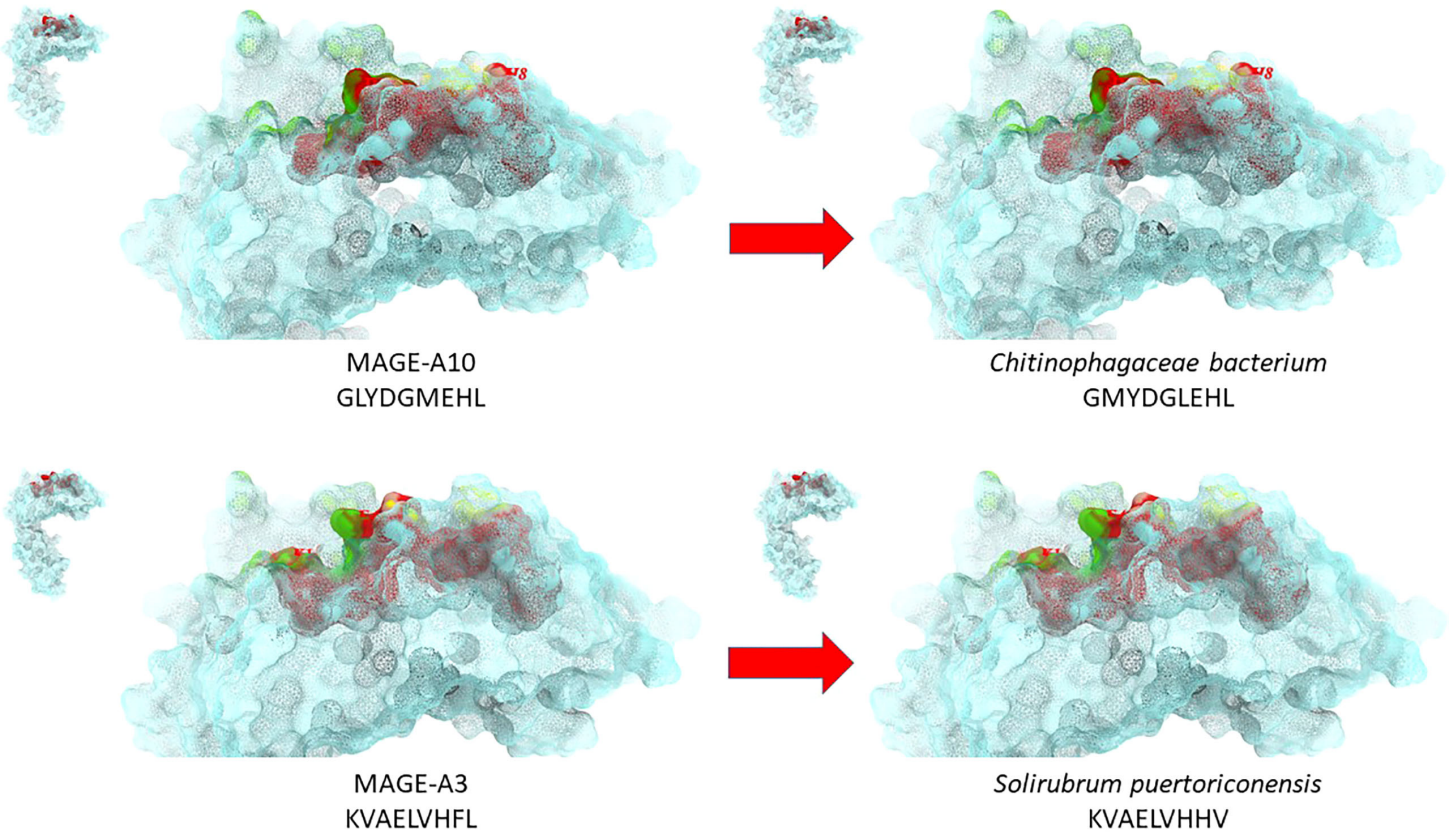
Structural predicted conformation of paired TAA and microbiota peptides. The conformation of the paired microbiota and TAA peptides bound to the HLA-A*02:01 molecule is shown. The prediction was performed using as template structure the HTLV-I LLFGYPVYV peptide crystallized with the HLA-A*0201 molecule, the β2 microglobulin, the α and β chains of the TCR (PDB https://www.rcsb.org/structure/1AO7). Green areas = contact points with the TCR α chain; Yellow areas = contact points with the TCR β chain.

More recently, the same analysis has been performed by submitting the TAAs from the cancer peptide database to BLAST for a protein homology search against microbiota Firmicutes (taxid:1239) and bacteroidetes (taxid:976) sequences. For the first time we have shown the high homology between TAAs and peptides derived from microbiota species which account for 90% of gut microbiota (19). About 80% of the paired epitopes show 6-8 identical residues along the 9-aa peptide sequence. The amino acid substitutions at each position are mostly conservative, confirming that the replacement does not significantly influence the charge and conformation of the peptide structure. Strikingly, three microbiota-derived peptides showed a sequence identical to the corresponding TAA, the GnTV (Firmicutes Oscillospiraceae bacterium Seq ID MBP0955392.1), MAGE-C1 (Firmicutes Clostridia bacterium Seq ID MBO5479965.1) and MK-NH-1 (Firmicutes Roseburia sp. 1XD42-69 Seq ID WP_120409454.1). The average predicted affinity of the microbiota-derived epitopes to the HLA-A molecules is extremely high (<100nm), strongly suggesting a validated binding to HLA molecules and efficient presentation to T cells. The structural conformation of the paired microbiota-derived epitopes and TAAs is very similar, if not identical, and contact areas with both HLA and TCR chains are indistinguishable (19) (**Figure 2**).

Notably, the homology has been found with the MAGE-A3 and MAGE-A10 TAAs overexpressed in several cancers of different histological origin, suggesting that it may have a strong impact on several cancers (20, 21).

## Discussion and future directions

The molecular mimicry performed by T cells elicited by viral and microbiota antigens able to cross-react with homologous tumor-associated antigens opens a very new horizon in cancer immunology and immunotherapy. Indeed, at individual level, the broader is the MoAs-specific memory immunity elicited during the lifetime by infections, the higher is the probability that memory CD8+ T cells would promptly cross-react to similar TAAs expressed by cancer cells. Ultimately, natural infections may represent an anti-cancer preventive immunization and the net result would be the elimination of cancer cells in the very early stages, preventing tumor from progressing.

Furthermore, MoAs with homology to TAAs represent a completely novel class of highly immunogenic universal antigens to be used in cancer immunotherapy (e.g. preventive/therapeutic cancer vaccines). Indeed, such non-self antigens are not affected by central immune tolerance and elicit potent T cell responses with anti-tumor effect.

Therefore, we strongly believe that the inclusion of such MoAs in cancer vaccine formulations should and will be actively pursued in the field in order to considerably boost the efficacy of anti-cancer immunotherapies.

## Data availability statement

The original contributions presented in the study are included in the article/Supplementary Material. Further inquiries can be directed to the corresponding author. Raw data are available at the public repository https://zenodo.org/record/7124328#.Yz7KQHbP2Uk.

## Author contributions

MT and LB conceived and drafted the perspective. All authors contributed to the article and approved the submitted version.

## Funding

The study was funded by the Italian Ministry of Health through Institutional “Ricerca Corrente” (MT & LB); POR FESR 2014/2020 “Campania OncoTerapie” (LB); POR FESR 2014/2020 “NanoCAN” (LB).

## Conflict of interest

The authors declare that the research was conducted in the absence of any commercial or financial relationships that could be construed as a potential conflict of interest.

## Publisher’s note

All claims expressed in this article are solely those of the authors and do not necessarily represent those of their affiliated organizations, or those of the publisher, the editors and the reviewers. Any product that may be evaluated in this article, or claim that may be made by its manufacturer, is not guaranteed or endorsed by the publisher.
